# Long-Term Empagliflozin Treatment (50 Days) Attenuates Protease Activity, Migration, and Stemness-Associated Phenotypes in BFTC-909 Renal Pelvis Transitional Cell Carcinoma Cells

**DOI:** 10.7150/jca.132959

**Published:** 2026-07-13

**Authors:** Yi-Hsun Lee, Pei-Ni Chen, Yi-Hsien Hsieh, Yi-Cheng Chu, Li-Jeng Chen, Chin-Yin Lin, Shun-Fa Yang, Horng-Rong Chang

**Affiliations:** 1 Institute of Medicine, Chung Shan Medical University, Taichung, Taiwan.; 2 Department of Medical Research, Chung Shan Medical University Hospital, Taichung, Taiwan.; 3 Division of Nephrology, Department of Internal Medicine, Chung Shan Medical University Hospital, Taichung, Taiwan.; 4 School of Medicine, Chung Shan Medical University, Taichung, Taiwan.

**Keywords:** empagliflozin, metastasis, invasion, matrix metalloproteinase-2, stemness-associated phenotypes

## Abstract

Empagliflozin is a sodium-glucose cotransporter 2 inhibitor widely prescribed for the treatment of type 2 diabetes mellitus. It promotes urinary glucose excretion by inhibiting renal glucose reabsorption. Although empagliflozin has been widely used in clinical practice, its effects and underlying mechanisms related to metastasis, cytokine secretion, and stemness-associated phenotypes in renal pelvis transitional cell carcinoma (TCC) remain poorly understood. In this study, we investigated the long-term (50 days) effects of empagliflozin (5 μM) on BFTC-909 human renal pelvis TCC cells. Prolonged empagliflozin treatment suppressed the migration of tumor cells and the proteolytic activities of matrix metalloproteinase-2 and urokinase-type plasminogen activator. In addition, empagliflozin markedly reduced the viability and self-renewal capacity of BFTC-909 cells. Analysis of conditioned media revealed that long-term empagliflozin exposure significantly reduced the secretion of interleukin-1Ra, interleukin-8, granulocyte colony-stimulating factor, and vascular endothelial growth factor. Together, these findings suggest that empagliflozin suppresses the protease activity, tumor invasiveness, and stemness-associated phenotypes of renal pelvis TCC cells. Overall, this study indicates that long-term empagliflozin treatment reduces the metastatic potential of BFTC-909 renal pelvis TCC cells and highlights the need for additional *in vivo* and clinical investigations.

## Introduction

Empagliflozin is a sodium-glucose cotransporter 2 (SGLT2) inhibitor and a oral hypoglycemic agent widely prescribed for type 2 diabetes mellitus (T2DM). The recommended adult dose is 10 mg once daily and may be increased to 25 mg daily. Empagliflozin primarily improves glycemic control by inhibiting SGLT2 activity in renal proximal tubular cells, thereby reducing glucose reabsorption and increasing urinary glucose excretion 1. SGLT2 is predominantly expressed in the renal proximal tubules and plays a vital role in maintaining blood glucose homeostasis by transporting filtered glucose back into circulation. In patients with T2DM, SGLT2 expression is upregulated, resulting in excessive glucose reabsorption and persistent hyperglycemia. Persistent hyperglycemia contributes to the development of cardiovascular and renal complications. Empagliflozin protects against renal tubular ferroptosis by activating the adenosine monophosphate-activated protein kinase (AMPK)/nuclear factor erythroid 2-related factor 2 pathway in diabetic kidney disease 2. It also alleviates cardiac microvascular ischemia by maintaining endothelial function and mitochondrial homeostasis through the suppression of Fis1-mediated mitochondrial fission 3.

Nonclinical data suggest that empagliflozin has a favorable safety profile and is not genotoxic 4. Evidence also suggests that empagliflozin induces renal tumor formation only in male mice at exposure levels far exceeding clinical doses through a nongenotoxic, cytotoxic, and proliferation-driven mechanism, indicating limited relevance to human safety 4. Empagliflozin itself is not cytotoxic or genotoxic to mouse renal tubular cells. However, a male mouse-specific oxidative metabolite of empagliflozin (M466/2) induces cytotoxicity and renal metabolic stress through the generation of reactive 4-OH crotonaldehyde. These findings support an indirect, nongenotoxic mechanism of renal tumor formation in male mice 5. Notably, long-term high-dose empagliflozin treatment (1000 mg/kg/day for 13 weeks) was demonstrated to increase Ki-67-positive proliferating cell count, p53 expression, and cell cycle activity in the renal tubular epithelial cells of male CD-1 mice 6.

Several studies have investigated the association between SGLT2 inhibitor use and cancer risk in patients with T2DM. Meta-analyses have reported that long-term treatment with SGLT2 inhibitors (for at least 24 weeks) does not substantially modulate the overall incidence of most cancers. However, empagliflozin treatment increases the risk of bladder cancer, whereas canagliflozin treatment reduces the risk of gastrointestinal cancers 7. The EMPA-REG OUTCOME trial demonstrated that empagliflozin treatment did not significantly increase the risk of transitional cell carcinoma (TCC) or bladder cancer in patients with T2DM 8. A study revealed that SGLT2, but not sodium-glucose cotransporter 1, is considerably upregulated in metastatic lung cancer lesions compared with its levels in primary tumors, suggesting that SGLT2 plays key roles in glucose uptake and metabolic adaptation during lung cancer metastasis 9. In pancreatic cancer xenograft models, SGLT2 inhibitors suppressed glucose uptake and inhibited tumor growth and survival, demonstrating their potential as repurposed anticancer agents 10. The use of SGLT2 inhibitors did not increase the risk of urothelial carcinoma (UC) at different anatomical sites of the urinary tract 11. In a 2-year CD-1 mouse study, empagliflozin boosted the development of renal tubular tumors only in male mice receiving high doses, but no such effects were observed in female mice 12. Despite these findings, the effects of long-term empagliflozin treatment on cancer progression in patients with renal pelvis TCC and concurrent T2DM remain unclear. Therefore, the present study investigated the long-term effects (50 days) of empagliflozin on urinary tract malignancies, focusing on cancer stem cell (CSC) characteristics, cell proliferation, and metastatic potential.

The urothelial epithelium is distributed throughout the urinary tract, extending from the renal pelvis and ureter to the bladder and the proximal two-thirds of the urethra. This epithelial layer lines the inner surface of the urinary tract, where it directly contacts urine and serves as a protective barrier for the underlying tissue 13. UC arises from the malignant transformation of urothelial epithelial cells and was historically referred to as TCC on the basis of its histopathologic characteristics. Metastasis is the primary cause of cancer recurrence, treatment failure, and cancer-related mortality.

The effects of long-term empagliflozin treatment on cell proliferation, metastasis, cytokine secretion, and CSC properties in renal pelvis TCC remain underexplored. Moreover, whether empagliflozin exerts tumor-promoting or tumor-suppressive effects during the development and progression of renal pelvis TCC remains unclear. Therefore, the present study investigated the effects of long-term empagliflozin exposure on cytokine secretion, CSC characteristics, and invasive potential in urinary tract malignancies.

## Materials and Methods

### Cell culture and empagliflozin treatment

BFTC-909 cells were purchased from Bioresource Collection and Research Center (Hsinchu, Taiwan) and were grown in Dulbecco's modified Eagle's medium (DMEM) supplemented with 10% (v/v) fetal bovine serum (FBS) and 1% penicillin/streptomycin. All cell cultures were maintained in a humidified incubator at 37 °C with 5% CO₂. For empagliflozin (Merck KGaA, Darmstadt, Germany; purity ≥ 98%, HPLC) treatment, an appropriate volume of stock solution (5 mM in dimethyl sulfoxide) was added to DMEM to achieve a final concentration of 5 μM. This concentration was selected based on previous *in vitro* studies, which commonly use low micromolar concentrations (1-5 μM) to evaluate biological effects while minimizing nonspecific cytotoxicity 14, 15. A 0.1% dimethyl sulfoxide solution was used as the vehicle control. In this study, a long-term treatment model (50 days) was established to better mimic the clinical use of empagliflozin, which is typically administered chronically in patients with type 2 diabetes. BFTC-909 cells were continuously treated with empagliflozin (5 μM) for 50 days to simulate sustained drug exposure and to evaluate its cumulative effects on cellular behavior. For the long-term treatment group, BFTC-909 cells (passages 12-20) were cultured in DMEM supplemented with 5 μM empagliflozin at a density of 8 × 10⁵ cells per 10-cm dish until reaching confluence. The cells were passaged every 3 days for a total of 50 days. Control cells were cultured in parallel under identical conditions without empagliflozin for the same duration.

### Assessment of invasion and motility using a Boyden chamber assay

After long-term treatment (50 days) with empagliflozin (0 or 5 μM), BFTC-909 cells were harvested and added to the upper chamber at a cell density of 1.5 × 10^4^ cells/well and incubated for an additional 24 h. For the invasion assay, polycarbonate membrane was coated with Matrigel® matrix (BD Biosciences, Bedford, MA) to provide a reconstituted extracellular matrix barrier. For the motility assay, the membrane was used without Matrigel coating. Following incubation, the invaded and migrated cells were fixed with methanol and stained with Giemsa stain. The number of invaded and migrated cells was quantified under an optical microscope.

### Wound healing assay for cell migration

After long-term treatment with empagliflozin (0 or 5 μM) for 50 days, BFTC-909 cells were seeded into six-well plates and cultured until they formed a confluent monolayer, followed by an additional 24 hours incubation. Wounds were generated using culture-inserts (Ibidi GmbH) to create a uniform cell-free gap. The cells were then exposed to DMEM containing 0.5% FBS for 24 hours. Migration of cells into the wound area was photographed using a phase-contrast microscope at 100× magnification.

### Zymography assay for determination of metalloproteinases-2 (MMP-2) and urokinase-type plasminogen activator (u-PA) activity

For gelatin zymography, collected conditioned media were loaded into the gelatin‒embedded polyacrylamide gels to evaluate MMP-2 activity. After electrophoresis, the gels were washed to remove SDS and incubated in reaction buffer at 37 °C to allow enzymatic digestion of the substrate. The gels were then stained with Coomassie brilliant blue and incubated with destaining solution until clear bands corresponding to proteolytic activity became visible. For casein zymography, gelatin in the gels was replaced with casein to determine the activity of u-PA. Quantitative data were analyzed using ImageJ software (National Institutes of Health, Bethesda, MD, USA) 16.

### Microculture tetrazolium (MTT) assay for cell proliferation

BFTC-909 and HK-2 cells were treated with empagliflozin (0 or 5 μM) for 50 days. Following treatment, the cells were seeded into 24-well plates at a density of 2 × 10⁴ cells per well and incubated for an additional 24, 48, and 72 h. The cells were then washed and incubated with 0.5 mg/ml 3-(4,5-dimethylthiazol-2-y1)-2,5-diphenyltetrazolium bromide (Sigma, St. Louis, MO) in culture medium. After 4 h incubation, the blue formazan crystals formed by viable cells were dissolved, and the absorbance was measured at 570 nm using a spectrophotometer 17.

### Western blot assay

BFTC-909 cells were treated with empagliflozin (0 or 5 μM) for 50 days. Total cell lysates from cells subjected to long-term empagliflozin treatment were collected, and equal amounts of extracted proteins were separated by 10% SDS-PAGE and transferred onto nitrocellulose membranes. The membranes were then incubated with specific primary antibodies, followed by appropriate horseradish peroxidase-conjugated secondary antibodies. The bound antibodies were visualized by chemiluminescence using a Luminescent Image Analyzer LAS-4000 mini (GE Healthcare) 18. After densitometric analysis of band intensities using ImageJ software (National Institutes of Health, Bethesda, MD, USA), relative protein levels were quantified by normalization to β-actin.

### Cell-matrix adhesion analysis

BFTC-909 cells subjected to long-term empagliflozin treatment were seeded into 24-well plates that had been pre-coated with either collagen type IV or collagen type I and allowed to adhere for 40 min. After incubation, nonadherent cells were removed by gently washing with PBS. The adherent cells were then fixed with methanol and stained with crystal violet. Subsequently, the bound dye was solubilized using 30% acetic acid, and the absorbance was measured at 550 nm using a spectrophotometer to quantify cell-matrix adhesion.

### Aldefluor assay and flow cytometry

BFTC-909 cells were treated with empagliflozin (0 or 5 μM) for 50 days. The activity of aldehyde dehydrogenase 1 (ALDH1) in BFTC-909 cells following long-term empagliflozin treatment was evaluated using the ALDEFLUOR assay kit. Briefly, 1 × 10^6^ cells were suspended in assay buffer, and the ALDEFLUOR™ substrate was added. Cells were first gated based on forward and side scatter to exclude debris, followed by doublet discrimination. For the negative control, diethylaminobenzaldehyde (DEAB), a specific inhibitor of ALDH1, was added to define background fluorescence. ALDH-positive cells were then defined using the DEAB-treated sample as a negative control to establish the gating threshold. After incubation, the cells were washed with PBS, resuspended in assay buffer. Cells were collected and analyzed using a BD FACSCanto ™ II flow cytometer. Data acquisition and analysis were performed using FlowJo™ v10.6.0 software 19.

### Sphere formation assay

BFTC-909 cells were treated with empagliflozin (0 or 5 μM) for 50 days. Following long-term treatment, cells were dissociated into single-cell suspensions, and 5 × 10³ cells were seeded into low-attachment six-well plates and cultured in tumor sphere medium consisting of serum-free DMEM/F12 supplemented with N2 supplement (R&D Systems), 20 ng/mL EGF, 20 ng/mL bFGF, and 1% penicillin/streptomycin (Hyclone). Cells were cultured and quantified for an additional 3, 6, and 9 days to allow tumor sphere formation (> 50 μm diameter).

### Bio-plex pro human cytokine assay

Cytokine profiles in the conditioned media from BFTC-909 cells subjected to long-term empagliflozin treatment were determined using a Bio-Plex Pro human cytokine 27-plex assay (Bio-Rad Laboratories). Briefly, assay buffer (100 μL) and antibody-coupled beads (50 μL) were added to each well of a 96-well assay plate. Subsequently, condition media was added to each well and incubated for 1 hour on a shaker in the dark, followed by three times with wash buffer to remove unbound substances. The detection antibodies were added to each well. After incubating with streptavidin-phycoerythrin solution, the antibody-coupled beads were resuspended in assay buffer. Cytokine concentrations were measured using a Bio-Plex 200 suspension array system (Bio-Rad) and quantified with Bio-Plex Manager™ software. Data obtained from the 27-plex assay were normalized to the number of viable cells to account for differences in cell viability.

### Glucose uptake assay

BFTC-909 cells were treated with empagliflozin (0 or 5 μM) for 50 days. Glucose uptake activity was determined using a Glucose Uptake Assay Kit (ab136955, Abcam, Cambridge, MA, USA). Following long-term treatment, cells were seeded into 6-well plates at a density of 1 × 10^5^ cells per well. After overnight starvation, cells were incubated with 10 μL of 10 mM 2-deoxyglucose (2-DG) for 20 minutes. Cells were then lysed, and the cooled cell lysate was mixed with assay buffer, followed by the addition of Reaction Mix A and incubation for 1 hour. After incubation, Neutralization Buffer II was added, followed by Reaction Mix B. The absorbance was measured at 412 nm using a microplate reader, and all results were normalized to cell viability.

### Statistical analysis

All experiments were performed with at least three independent biological replicates, and data are presented as mean ± SD. Statistical comparisons between the vehicle control and empagliflozin-treated groups were performed using Student's t-test. For experiments involving multiple time points, including the MTT assay (24, 48, and 72 h) and sphere formation assay (3, 6, and 9 days), comparisons were performed between the control and empagliflozin-treated groups at each individual time point. Quantitative data obtained from the migration and invasion assays, zymography, Western blotting, cell-matrix adhesion assay, ALDEFLUOR assay, glucose uptake assay, and cytokine analysis were analyzed in the same manner. A *P* value < 0.05 was considered statistically significant.

## Results

### Long-term empagliflozin treatment reduces cell viability and modulates cell cycle-related protein expression in BFTC-909 cells

**Figure 1:**
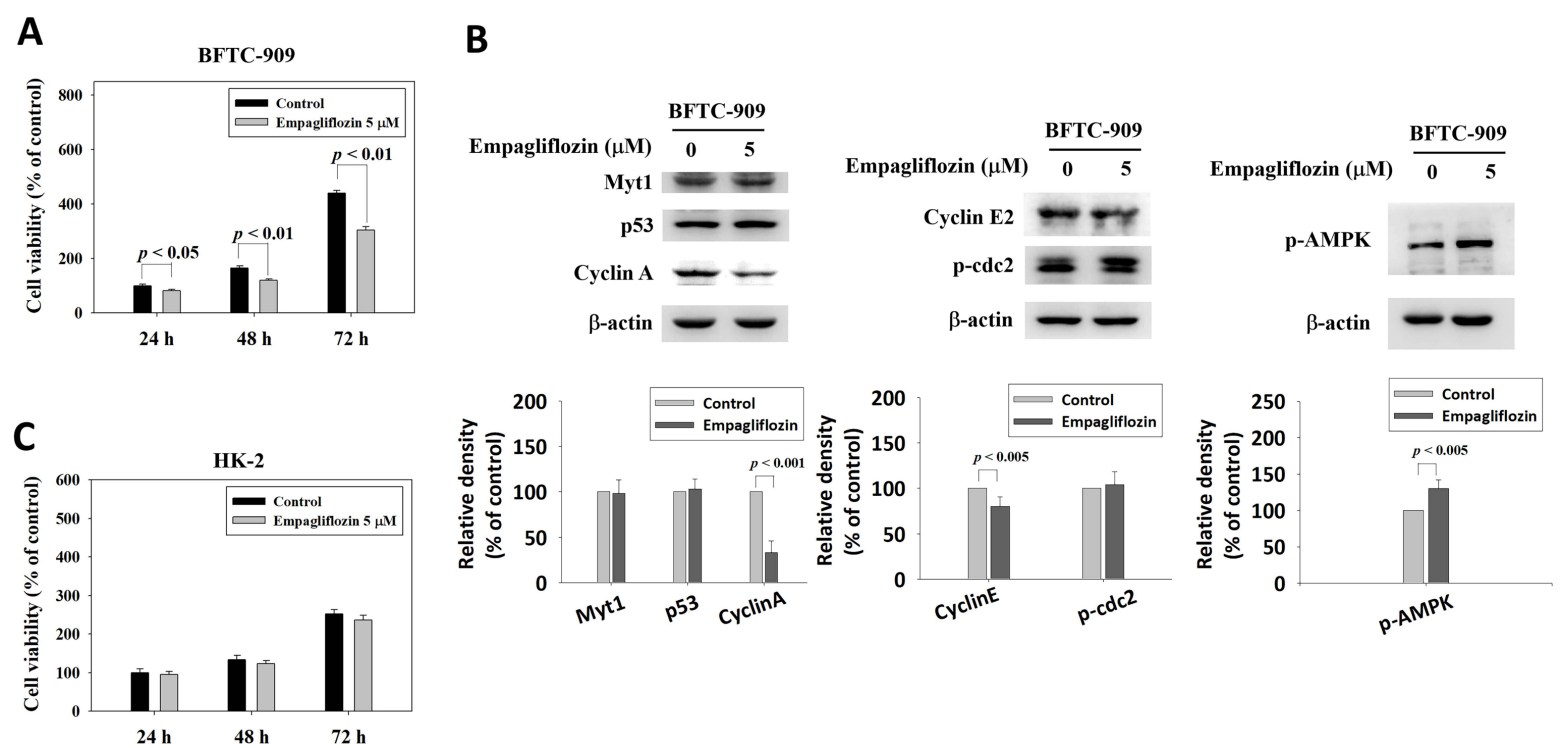
Inhibitory effects of long-term treatment of empagliflozin on viability and cyclin A expression in BFTC-909 cells. (A) BFTC-909 cells were treated with empagliflozin (0 or 5 μM) or vehicle control for 50 days, and cell viability was determined by the MTT assay. (B) Cell lysates of BFTC-909 were determined the protein expression of Myt1, p53, cyclin A, cyclinE2, p-cdc2, and p-AMPK by Western blot. (C) HK-2 cells were incubated with empagliflozin or vehicle control for 50 days and determined using MTT test. Quantitative data are presented as the mean ± SD from three independent experiments and were analyzed using Student's *t*-test.

The viability of BFTC-909 cells was evaluated over time by using the MTT assay after long-term empagliflozin treatment (5 μM for 50 days). After additional treatment for 24, 48, and 72 h, empagliflozin significantly reduced cell viability; this finding suggests that empagliflozin inhibits the growth of BFTC-909 cells (Figure 1A).

To investigate the effects of empagliflozin on cell cycle-related proteins, the expression levels of Myt1, p53, phosphorylated cdc2 [p-cdc2 (Tyr15)], Cyclin A, and Cyclin E2 were analyzed through Western blotting, using β-actin as the loading control. Empagliflozin treatment (5 μM) did not significantly modulate the expression level of Myt1, p53, or p-cdc2 (Tyr15) in BFTC-909 cells. However, a marked reduction was noted in Cyclin A level, suggesting that empagliflozin affects S/G2 phase cell cycle regulation. Cyclin E2 expression was slightly downregulated after empagliflozin treatment (Figure 1B).

To identify the upstream pathway associated with the downregulation of Cyclin A in BFTC-909 cells, phosphorylated AMPK (p-AMPK) levels were analyzed. Empagliflozin treatment increased the level of p-AMPK (Figure 1B), suggesting activation of the AMPK pathway. These findings highlight a potential association between AMPK activation and Cyclin A downregulation. The MTT assay revealed that empagliflozin exerted no significant cytotoxic effects on nonmalignant human proximal tubule epithelial HK-2 cells (Figure 1C).

### Inhibitory effects of empagliflozin on the migration of BFTC-909 cells

**Figure 2:**
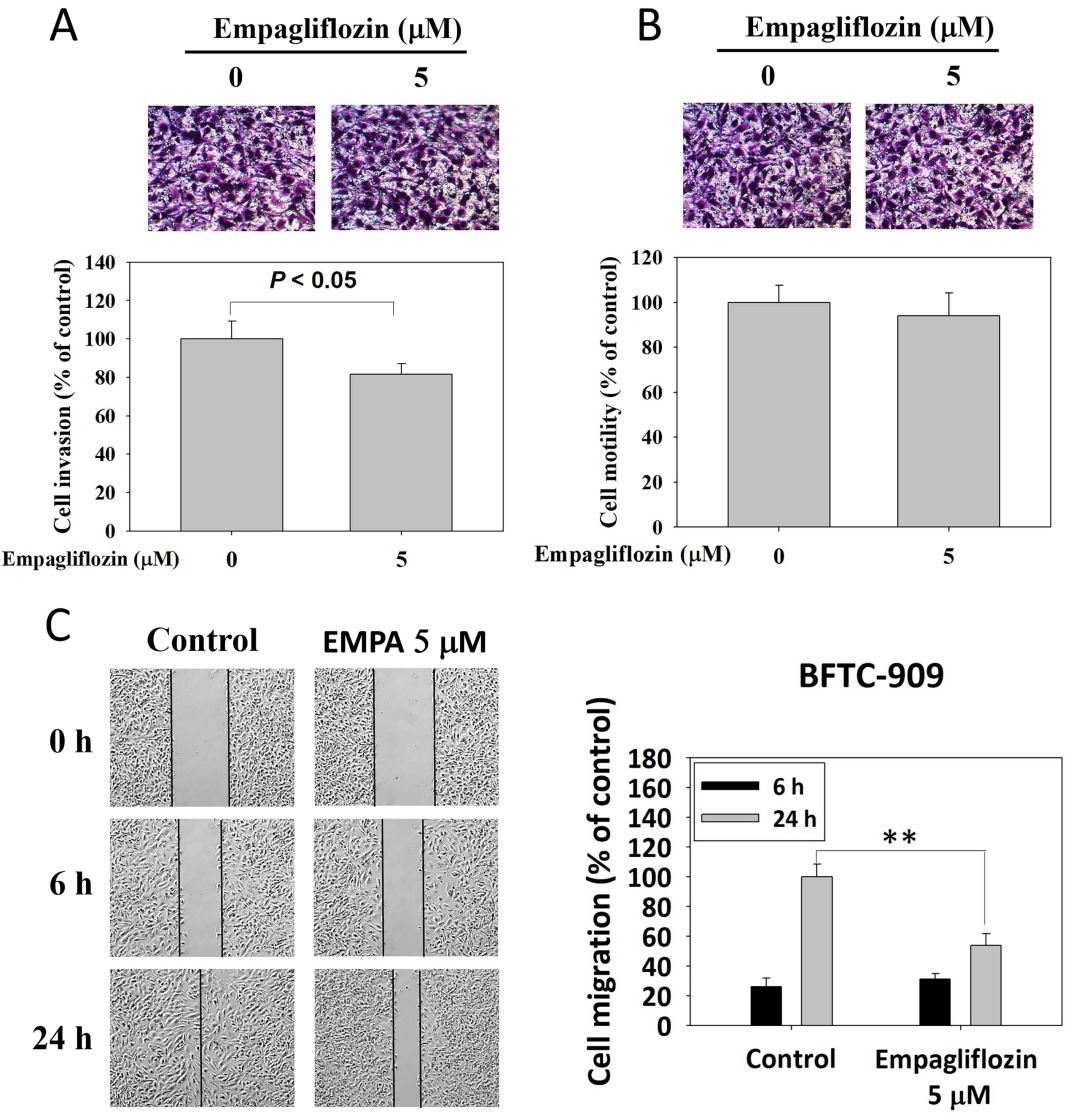
Effects of long-term treatment of empagliflozin on migration, motility, and invasiveness in BFTC-909 cells. Long-term treatment of empagliflozin (EMPA) BFTC-909 cells were subjected to invasion assay, motility assay and wound healing assays to determine the invasion, motility, and migration ability, respectively. Quantitative data are presented as the mean ± SD of three independent experiments and were analyzed using Student's *t*-test.

The Matrigel invasion assay revealed that empagliflozin (5 μM)-treated BFTC-909 cells exhibited slightly invasiveness than did control cells (Figure 2A). However, no significant difference was observed in cell motility between the treatment and control groups, indicating that empagliflozin exerted a limited effect on the intrinsic motility of BFTC-909 cells (Figure 2B). Cell migration after long-term empagliflozin treatment was evaluated using a wound healing assay. At 24 h, control cells significantly migrated toward the center of the wound area, whereas treated cells exhibited a marked delay in wound healing. These findings indicated that empagliflozin suppressed the migratory ability of BFTC-909 cells (Figure 2C).

### Long-term empagliflozin treatment suppresses protease activity in BFTC-909 cells

**Figure 3:**
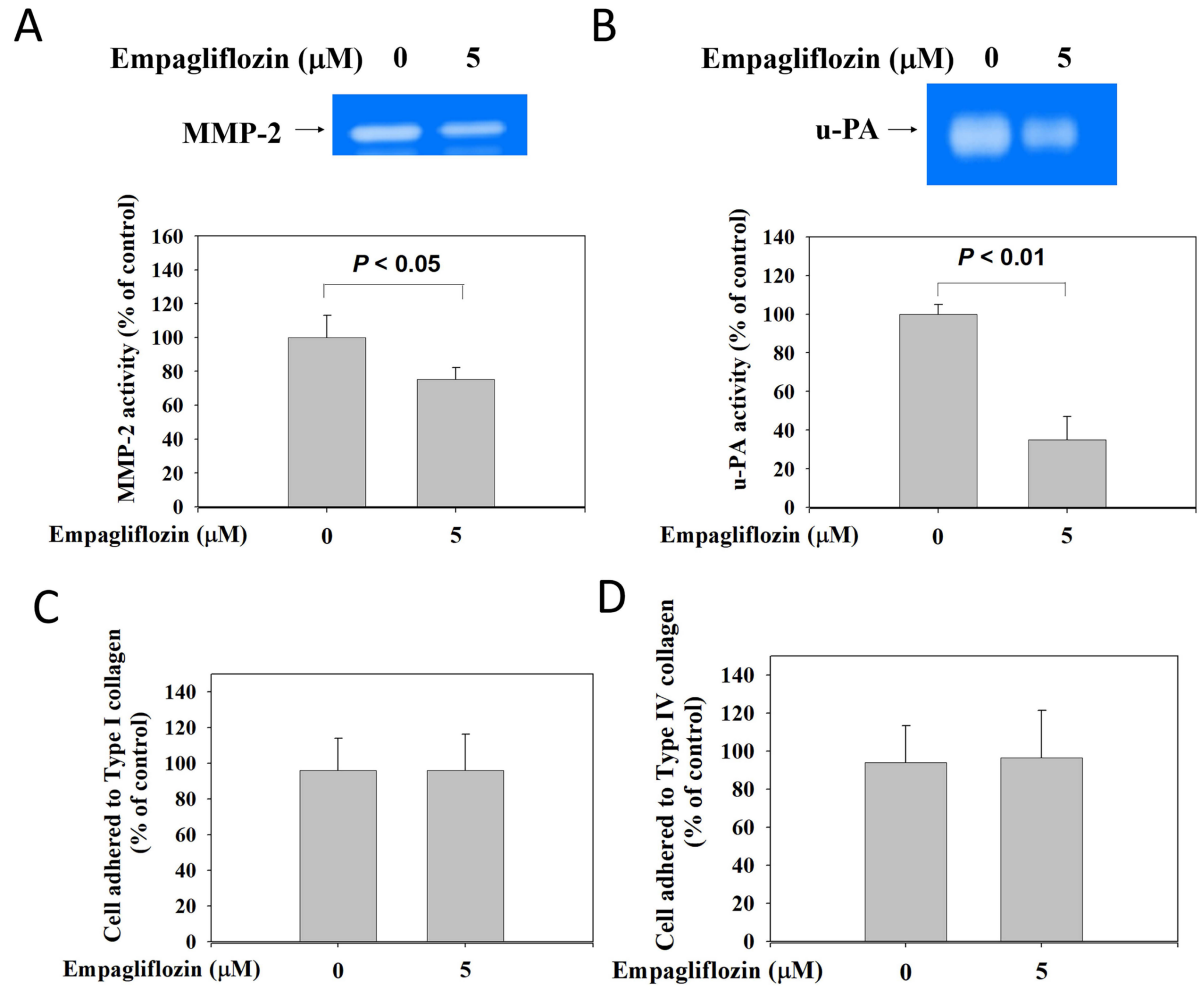
Inhibitory effects of long-term treatment of empagliflozin on proteinase activity in BFTC-909 cells. FBS-free conditioned media from long-term treatment with empagliflozin BFTC-909 cells were analyzed using a zymography system, with clear regions corresponding to (A) MMP-2 and (B) u-PA activity. Cell-matrix adhesion were used to detect the (C) cell-type I collagen and (D) cell-type IV collagen adhesion ability. Similar results were obtained from three independent experiments. Data are presented as mean ± SD and statistical significance was analyzed using Student's t-test.

After long-term empagliflozin (5 μM) treatment, BFTC-909 cells exhibited a marked reduction in the gelatin-degrading activity of MMP-2 in the conditioned medium (Figure 3A). Casein zymography analysis revealed that the treatment significantly reduced the proteolytic activity of u-PA (Figure 3B), indicating that empagliflozin suppresses the activity of proteolytic enzymes involved in tumor cell invasion. Cell adhesion to types I and IV collagen was evaluated using a cell-matrix adhesion assay. Empagliflozin treatment did not significantly alter cell adhesion to type I (Figure 3C) or IV (Figure 3D) collagen.

### Inhibitory effects of empagliflozin on fibronectin expression in BFTC-909 cells

**Figure 4:**
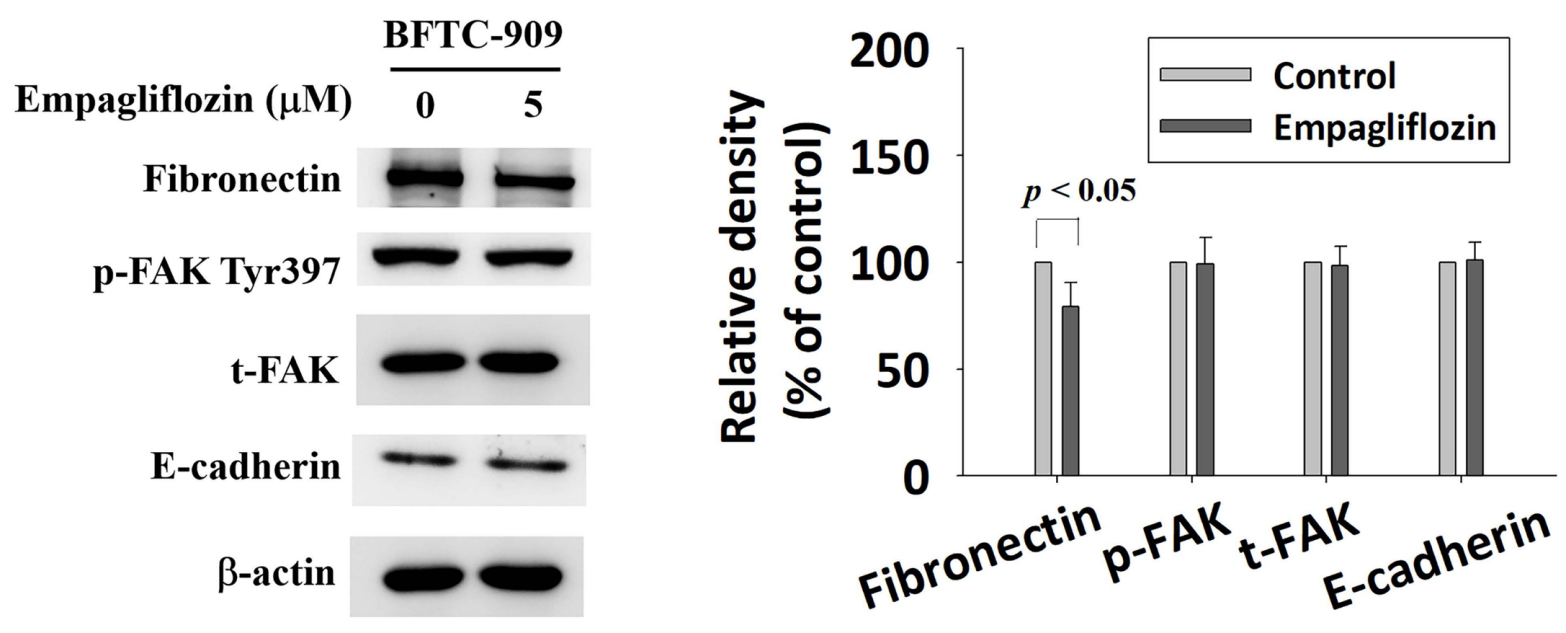
Effect of long-term treatment of empagliflozin on expression of EMT relative protein in BFTC-909. Cells were treated with empagliflozin or vehicle control and cell lysates were used to detect EMT related protein fibronectin, p-FAK, total-FAK, and E-cadherin by Western blot. Quantitative data are presented as the mean ± SD from three independent experiments and were analyzed using Student's *t*-test.

Biomarkers of epithelial-mesenchymal transition—fibronectin, phosphorylated focal adhesion kinase (FAK), total FAK, and E-cadherin—were analyzed through Western blotting. Empagliflozin treatment reduced fibronectin level by 21% (*P* < 0.05). However, it exerted no significant effect on the level of phosphorylated FAK, total FAK, or E-cadherin (Figure 4).

### Long-term empagliflozin treatment suppresses stemness-associated phenotypes in BFTC-909 cells

**Figure 5:**
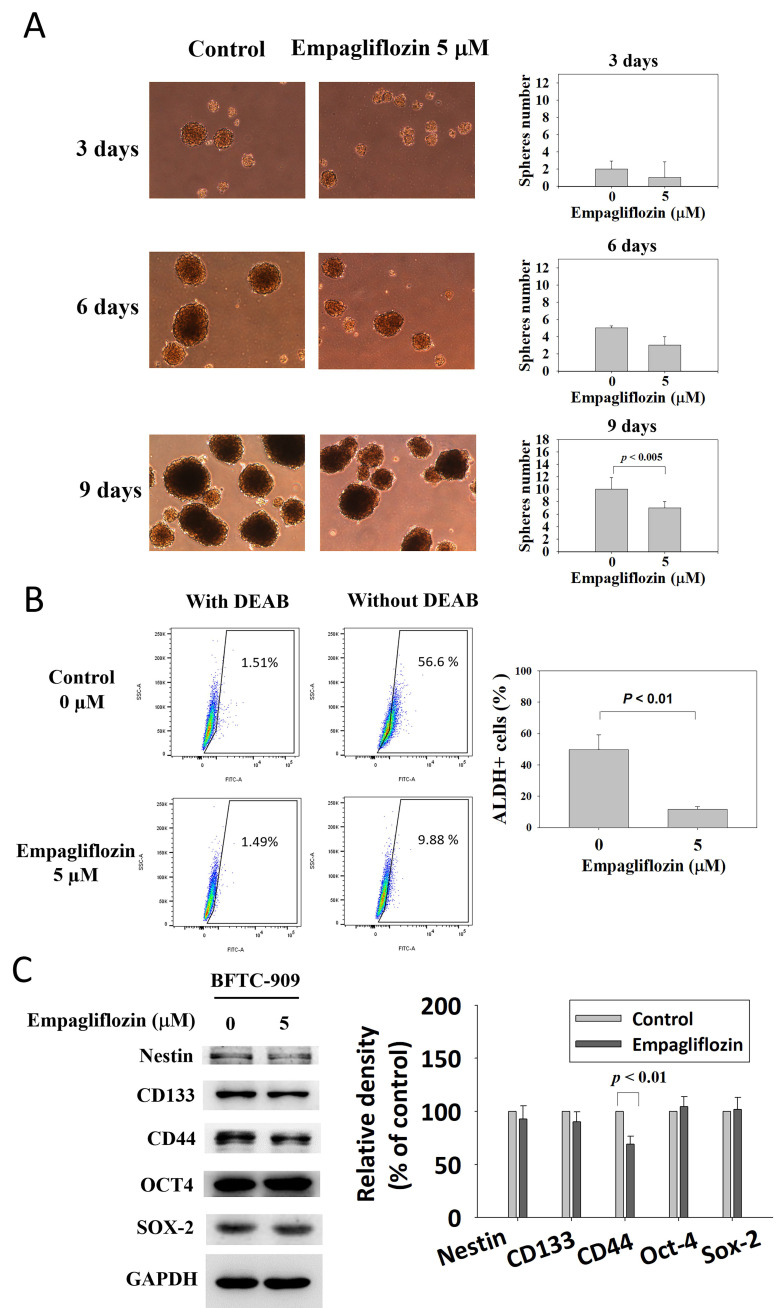
Suppression of self-renewal and CD44 expression of BFTC-909 cells by long-term treatment of empagliflozin. (A) Long-term treatment with empagliflozin BFTC-909 cells were plated onto low-attachment plates, and sphere formation were identified at 3, 6 and 9 days. (B) ALDH-bright BFTC-909 cells were determined using the Aldefluor assay. (C) The protein expression of Nestin, CD133, CD44, OCT4, and SOX-2 were detected by Western blot. Data are presented as mean ± SD from at three independent experiments.

After long-term empagliflozin treatment (5 μM), the number of tumor spheres formed by BFTC-909 cells decreased significantly, as indicated by the sphere formation assay (Figure 5A). The proportion of viable ALDH^+^ cells was determined using the ALDEFLUOR assay. Long-term empagliflozin treatment markedly reduced the percentage of ALDH^+^ BFTC-909 cells (control group vs. treatment group: 49.60% ± 9.5% vs. 11.59% ± 1.8%; Figure 5B). Representative gating plots are presented in Supplementary Figure S1. Western blotting revealed that long-term empagliflozin treatment significantly downregulated CD44 but exerted no significant effect on Nestin, CD133, OCT4, or SOX2 (Figure 5C).

### Long-term empagliflozin treatment suppresses cytokine secretion in BFTC-909 cells

**Figure 6:**
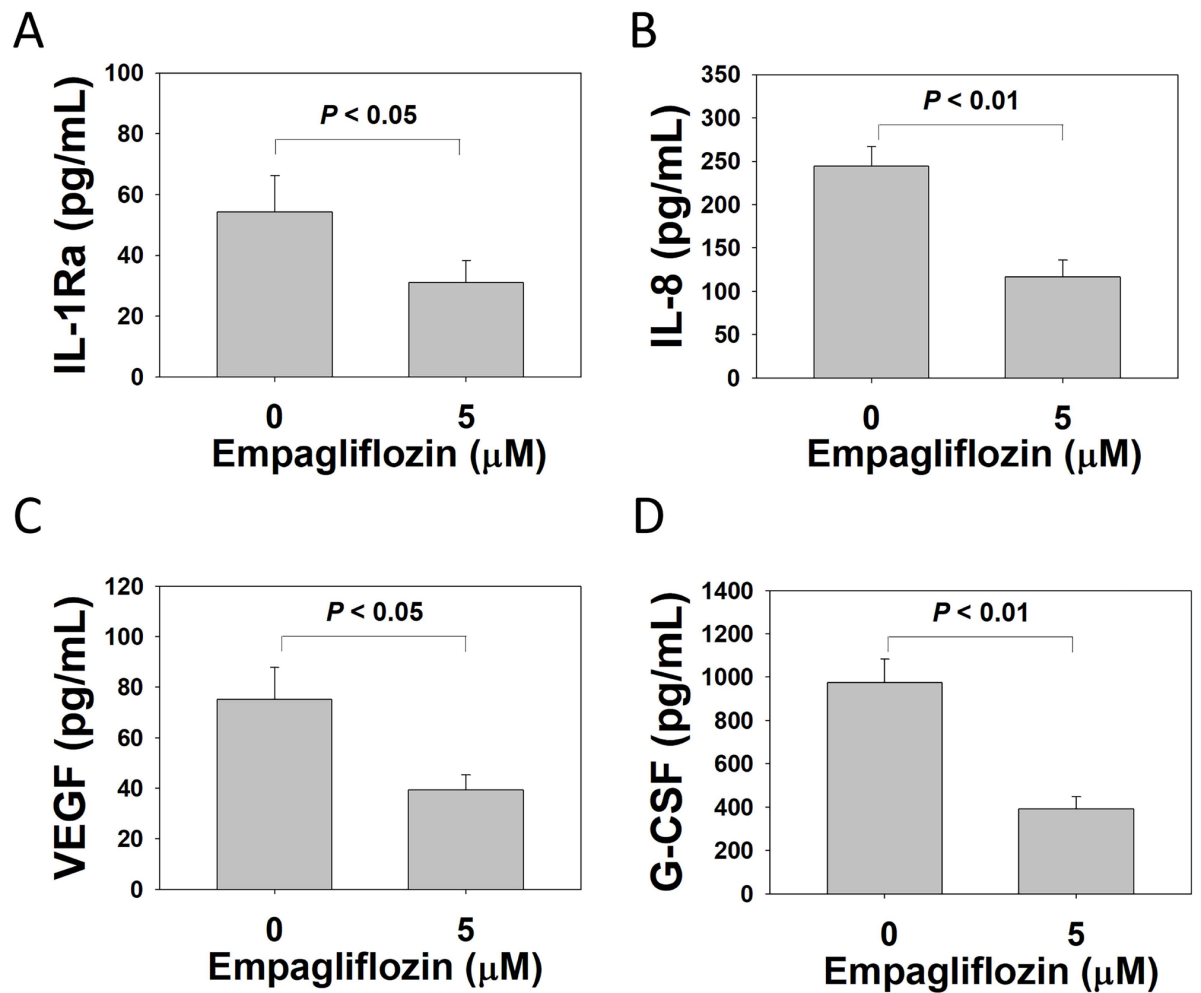
Suppression of IL-1Ra, IL-8, G-CSF, and VEGF of BFTC-909 cells by long-term treatment of empagliflozin. Using a 27-Plex cytokine analysis, BFTC 909 cells were treated with empagliflozin (0 or 5 μM) for 50 days. The culture medium was analyzed by cytokine array to examine the secretion of levels of (A) IL-1Ra, (B) IL-8, (C) VEGF and (D) G-CSF. Quantitative data are presented as the mean ± SD of three independent experiments and were analyzed using Student's *t*-test.

After long-term treatment with empagliflozin (5 μM), cytokine secretion by BFTC-909 cells was evaluated using a 27-plex cytokine assay. Empagliflozin treatment significantly reduced the levels of interleukin (IL)-1Ra (control group vs. treatment group: 54.24 ± 11.99 vs. 31.05 ± 7.19 pg/mL), IL-8 (244.23 ± 23.00 vs. 116.87 ± 19.15 pg/mL), granulocyte colony-stimulating factor (G-CSF; 919.16 ± 109.69 vs. 390.31 ± 57.06 pg/mL), and vascular endothelial growth factor (VEGF; 75.12 ± 12.72 vs. 39.27 ± 6.01 pg/mL) in the culture medium (Figure 6). The complete cytokine dataset, including the expression levels of the remaining 23 analytes, is presented in Supplementary Table S1.

### Long-term empagliflozin treatment suppresses glucose uptake in BFTC-909 cells

To determine whether empagliflozin inhibits SGLT2 activity, a glucose uptake assay was performed to measure intracellular glucose levels in BFTC-909 cells. The results revealed that long-term empagliflozin treatment significantly reduced glucose uptake in BFTC-909 cells (control group vs. treatment group: 75.90 ± 6.65 vs. 49.46 ± 8.32 pmol/μL; Supplementary Figure S2A). To investigate the effects of long-term empagliflozin treatment on SGLT2 protein expression, SGLT2 levels were analyzed in BFTC-909 cells. Empagliflozin treatment did not significantly alter SGLT2 expression (Supplementary Figure S2B).

## Discussion

In patients with T2DM, empagliflozin increases the risk of bladder cancer (odds ratio: 4.49; 95% confidence interval: 1.21-16.73) 7. SGLT2 inhibitors may possess anticancer properties and can be repurposed as anticancer agents 20. In the present study, empagliflozin treatment reduced protease activity in BFTC-909 renal pelvis TCC cells. In addition, it suppressed invasion and stemness-associated phenotypes.

Metastatic dissemination is a complex, multistep process that involves substantial alterations in cellular physiology 21. These alterations include disruption of cell-cell adhesion and interactions between tumor cells and the extracellular matrix (ECM), enhancement of migratory and invasive capacities, reorganization of the cytoskeletal structure, and increased secretion of proteolytic enzymes such as MMPs and u-PA 22, 23. Proteases promote ECM degradation, thereby helping tumor cells invade surrounding tissues. Cancer cells subsequently intravasate into blood or lymphatic vessels, evade immune surveillance, adhere to endothelial cells, and extravasate into distant organs. After dissemination, tumor cells continue to proliferate, induce angiogenesis, and establish secondary tumors. These processes allow cancer cells to compete with normal tissues for nutrients and oxygen, leading to organ dysfunction and cancer-related mortality 24. In the present study, long-term empagliflozin treatment reduced the activity of u-PA and MMP-2 and suppressed the migratory and invasive abilities of BFTC-909 cells.

The differential effects observed across the assays may be attributable to distinct biological processes evaluated by each method. In the present study, empagliflozin did not significantly affect cell motility in the Boyden chamber assay performed without Matrigel, which primarily reflects the intrinsic migratory ability of individual cells. This finding suggests that empagliflozin does not directly impair basal single-cell movement. By contrast, a modest reduction in cell invasion was observed in the presence of Matrigel, indicating that empagliflozin interferes with ECM degradation and penetration. This finding is consistent with our results indicating reductions in the activity of MMP-2 and u-PA, both of which play key roles in matrix remodeling and invasive behavior 25. The wound healing assay revealed a significant inhibition of migration. Unlike the Boyden chamber assay, the wound healing assay reflects collective cell migration, which involves coordinated cell movement, cell-cell interactions, cytoskeletal dynamics, and, to some extent, cell proliferation. Therefore, the stronger inhibitory effect observed in the wound healing assay suggests that empagliflozin preferentially disrupts coordinated multicellular migration rather than intrinsic individual cell motility. We further noted that empagliflozin treatment did not significantly alter cell-matrix adhesion. This finding is consistent with the unchanged expression of phosphorylated FAK, a key regulator of integrin-mediated adhesion signaling 26. These findings suggest that empagliflozin does not markedly affect adhesion-related signaling in BFTC-909 cells. By contrast, empagliflozin treatment suppressed cell migration, indicating that cell-matrix adhesion was not the rate-limiting step for migration in this experiment. Together, these results indicate that empagliflozin does not significantly affect intrinsic cell motility but suppresses invasion and collective migration, potentially by inhibiting protease activity, modulating tumor microenvironment-related processes, and reducing cell proliferation.

CSCs, also known as cancer-initiating cells, have been detected in various solid tumors 27. These cells are characterized by self-renewal capacity, tumor-initiating ability, and resistance to conventional therapies. Common CSC markers include ALDH 28; transcription factors such as SOX2, NANOG, and OCT4; and cell surface markers such as CD133, CD117, CD44, and Nestin 29. In addition, metastasis-initiating cells have been isolated from circulating tumor cells in renal cancer and are strongly associated with metastatic progression 30. High expression levels of the CSC markers CD133 and CD44 in renal cell carcinoma are associated with poor prognosis, and these markers may serve as therapeutic targets 31. In the present study, empagliflozin treatment significantly suppressed the expression of CD44, but not Nestin, CD133, OCT4, or SOX2. These findings suggest that empagliflozin selectively affects specific stemness-associated pathways rather than broadly suppressing all CSC markers. CD44 regulates glycolysis and glucose uptake in cancer cells, both of which are closely associated with cancer stem-like properties 32. Therefore, the observed downregulation of CD44 expression may be attributable to the empagliflozin-induced reduction in glucose uptake. We further noted that empagliflozin attenuated stemness-associated phenotypes, as indicated by reductions in the sphere-forming capacity, ALDH1 activity, and CD44 expression of BFTC-909 cells.

Long-term empagliflozin treatment reduced glucose uptake in BFTC-909 cells, suggesting altered cellular glucose metabolism. However, whether these effects were mediated directly by SGLT2-dependent mechanisms or by additional off-target metabolic pathways requires further investigation. Enhanced glucose metabolism is a hallmark of cancer progression 33. Therefore, reduced intracellular glucose availability might have contributed to the suppression of invasion and stemness-associated phenotypes. Collectively, these findings suggest that the anticancer effects of empagliflozin may be associated, at least in part, with modulation of cellular metabolic pathways.

Tumor-associated inflammation plays a vital role throughout tumorigenesis by promoting epigenetic alterations, intensifying antiapoptotic signaling, stimulating cancer cell proliferation, inducing angiogenesis, and facilitating metastasis 34. Tumor-associated macrophages contribute to these processes through the secretion of VEGF to promote angiogenesis, IL-8 to promote cancer cell proliferation, G-CSF to promote invasive UC progression, and MMPs to remodel the ECM 35, 36. In the present study, long-term empagliflozin treatment reduced the secretion of select protumorigenic cytokines—IL-1Ra, IL-8, G-CSF, and VEGF—and suppressed MMP-2 activity in BFTC-909 cells. These findings suggest that empagliflozin modulates specific tumor-associated inflammatory pathways.

Long-term empagliflozin treatment may exert different effects from short-term exposure because prolonged treatment can induce cumulative cellular responses. Short-term treatment (e.g., 24-h exposure) primarily reflects acute cellular responses, whereas prolonged exposure may lead to gradual alterations in cellular metabolism and downstream signaling. These alterations might have contributed to the observed modulation of invasion and stemness-associated phenotypes, which were less apparent under short-term treatment conditions. Our long-term treatment model was designed to conceptually mimic the chronic clinical administration of empagliflozin in patients with T2DM and may therefore provide valuable insights into the potential effects of prolonged drug exposure. To minimize culture-related artifacts, control cells were cultured in parallel under identical conditions. However, prolonged *in vitro* culture may introduce clonal selection or phenotypic drift; therefore, the observed phenotypic changes require further validation. Most studies investigating the association between SGLT2 inhibitors and cancer have focused on bladder TCC 8. However, renal pelvis and bladder TCC share similar urothelial origins and molecular characteristics. Therefore, findings obtained using BFTC-909 cells may provide relevant insights into UC biology.

This study has some limitations. The findings are based solely on *in vitro* evidence obtained using a single cell line. Future studies using additional upper urinary tract UC cell lines are required to validate the generalizability of these findings. Furthermore, additional studies are required to determine whether long-term empagliflozin treatment can suppress tumor growth and metastasis in animal models of renal pelvis TCC.

In conclusion, to the best our knowledge, this study is the first to demonstrate that long-term empagliflozin treatment in BFTC-909 cells reduced protease activity, suppressed cell migration, and modulated stemness-associated phenotypes, including sphere formation and ALDH1 activity. In addition, empagliflozin reduced the secretion of select cytokines—IL-1Ra, IL-8, G-CSF, and VEGF. These findings provide preliminary evidence that long-term empagliflozin treatment can modulate malignant phenotypes in BFTC-909 renal pelvis TCC cells. However, further validation in additional *in vitro* (UC cells) and *in vivo* models is required.

## Supplementary Material

Supplementary figures and table.
