# Supplementary material for: Long-Term Empagliflozin Treatment (50 Days) Attenuates Protease Activity, Migration, and Stemness-Associated Phenotypes in BFTC-909 Renal Pelvis Transitional Cell Carcinoma Cells
Source: J Cancer. 2026 Jul 13;17(7):1373–83. doi: 10.7150/jca.132959 (PMC13410780; doi:10.7150/jca.132959)
Supplement: Supplementary file 1 — Supplementary figures and table. [file jcav17p1373s1.pdf]

Long-Term Empagliflozin Treatment (50 Days) Attenuates Protease Activity, Migration, and Stemness-Associated Phenotypes in Renal Pelvis Transitional Cell Carcinoma BFTC-909 Cells

Yi-Hsun Lee, Pei-Ni Chen, Yi-Hsien Hsieh, Yi-Cheng Chu, Li-Jeng Chen, Chin-Yin Lin, Shun-Fa Yang*, Horng-Rong Chang*

Supplementary Table S1. Cytokine Secretion Profiles in BFTC-909 Cells Following Long-Term Empagliflozin Treatment

Cytokine (pg/mL)	Empagliflozin (0 μM)	Empagliflozin (5 μM)
IL-1β	< OOR	< OOR
IL-2	< OOR	< OOR
IL-4	< OOR	< OOR
IL-5	18.31 ± 5.32	13.53 ± 6.21
IL-6	2387.87 ± 144.01	2106.31 ± 152.41
IL-7	< OOR	< OOR
IL-9	2.7 ± 1.87	1.13 ± 0.94
IL-10	< OOR	< OOR
IL-12	< OOR	< OOR
IL-13	< OOR	< OOR
IL-15	26.4 ± 4.21	26.4 ± 3.82
IL-17A	< OOR	< OOR
Eotaxin	0.62 ± 0.22	0.33 ± 0.30
Basic FGF	6.91 ± 2.84	5.52 ± 2.62
GM-CSF	6.04 ± 3.25	5.69 ± 2.74
IFN-γ	1.04 ± 0.14	1.03 ± 0.22
IP-10)	< OOR	< OOR
MCP-1 (MCAF)	13.46 ± 4.50	6.83 ± 3.93
MIP-1α	< OOR	< OOR
MIP-1β	3.47 ± 2.01	1.92 ± 0.88
PDGF-BB	< OOR	< OOR
RANTES	11.05 ± 4.24	11.38 ± 3.02
TNF-α	9.64 ± 3.16	10.42 ± 4.21

OOR: out of range

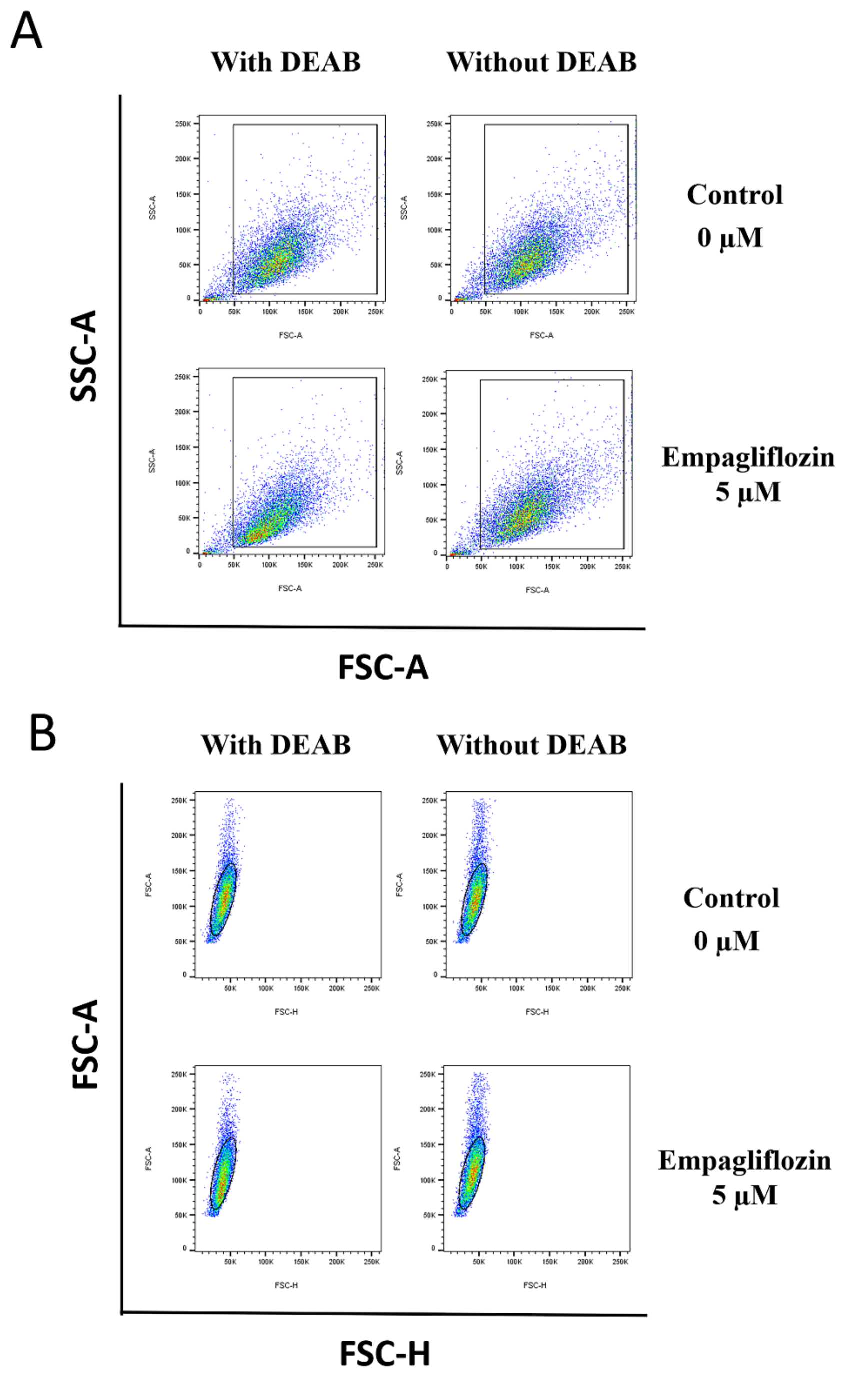

Supplementary Figure S1. Flow cytometry gating strategy for ALDEFLUOR assay. (A) Cells were gated based on side scatter (SSC) and forward scatter (FSC) to exclude debris. (B) Doublets and cell aggregates were then excluded by gating on FSC-A versus FSC-H to select single cells. $ALDH^+$ cells were subsequently identified based on fluorescence intensity using the ALDEFLUOR assay, with the DEAB-treated sample serving as a negative control to define the gating threshold.

A

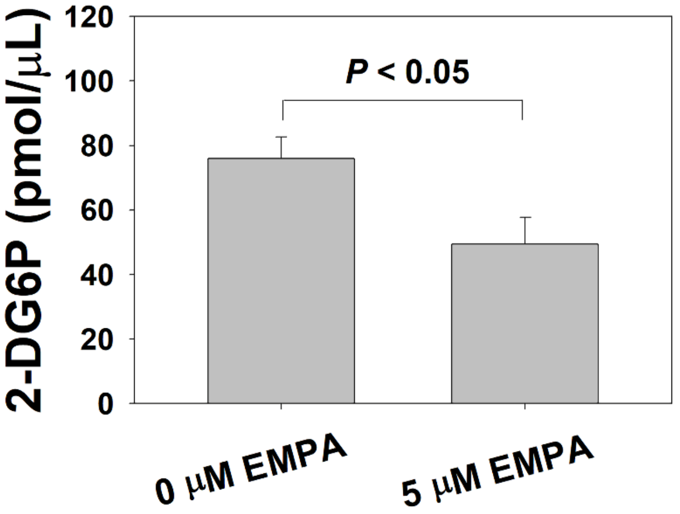

B

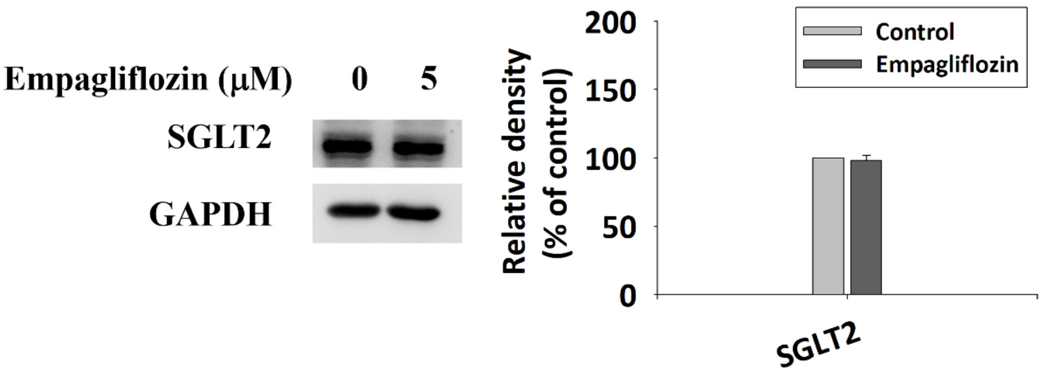

Supplementary Figure S2. Effects of long-term empagliflozin treatment on glucose uptake and SGLT2 expression in BFTC-909 cells. BFTC-909 cells were subjected to long-term treatment with 0 or 5 μM empagliflozin (EMPA) for 50 days. (A) Intracellular glucose levels were assessed using a glucose uptake assay. (B) Protein expression of SGLT2 in BFTC-909 cell lysates was analyzed by Western blotting using GAPDH as the loading control. Data are presented as mean ± SD from at least three independent experiments.
